# A Unique Case of a Gigantic Left Ventricular Myxoma Resulting in Embolic Acute Lower Limb Ischemia in a Pediatric Patient

**DOI:** 10.3390/jcm13082189

**Published:** 2024-04-10

**Authors:** Irina Margarint, Adelina Sorescu, Monica Popescu, Mircea Robu, Olga Untaru, Cristina Filip

**Affiliations:** 1 Faculty of Medicine, Carol Davila University of Medicine, and Pharmacy, 050474 Bucharest, Romania; irina-maria.margarint@drd.umfcd.ro (I.M.); cristina.filip@umfcd.ro (C.F.); 2 Department of Cardiac Surgery, Emergency Clinical Hospital for Children “Maria Skłodowska Curie”, 077120 Bucharest, Romania; untaru_olguta@yahoo.com; 3 Department of Pediatric Cardiology, Emergency Clinical Hospital for Children “Maria Skłodowska Curie”, 077120 Bucharest, Romania; 4 Department of Anesthesia and Intensive Care, Emergency Clinical Hospital Bucharest, 014461 Bucharest, Romania; doc.monica.popescu@gmail.com; 5 Prof. Dr. C.C. Iliescu Emergency Institute for Cardiovascular Diseases, 022322 Bucharest, Romania

**Keywords:** cardiac myxomas, lower leg ischemia, peripheral embolism, pediatric population

## Abstract

Background: The presence of a primary cardiac tumor in a pediatric patient is a rare echocardiographic finding. Case Report: We report the case of an 11-year-old female patient with multiple peripheral embolisms, due to a gigantic left ventricular tumor, with a unique echocardiographic appearance. The patient was referred to the emergency department due to acute pain and loss of sensitivity in both of her legs. Past medical history was significant for acute lymphoblastic leukemia. Upon physical examination, suspicion of bilateral lower leg ischemia was raised. Doppler arterial ultrasound of both legs confirmed the suspicion mentioned above, as the right lower extremity suffered from partial arterial occlusion of the external iliac artery and total occlusion of the femoral arteries. Meanwhile, in the left lower extremity, the occlusion was localized in the proximal tibio-peroneal artery. Cardiac sonography revealed a massive, mobile, left ventricular intracavitary mass. Aside from its large dimensions (6.3 cm by 3 cm), its aspect was striking as well as it had very mobile and friable edges. Emergency bilateral endarterectomy and excision of the left ventricular tumor were performed alongside systemic anticoagulant therapy, with excellent results, as no tumoral residual masses could be seen in the left ventricle, and the arterial blood flow was restored completely in both lower extremities. The histopathological aspect of the excised masses was that of a myxoma. The patient recovered well after surgery and was discharged on postoperative day 14. Conclusion: Despite only a handful of cases of cardiac myxomas being reported due to their rarity in the pediatric population, clinical presentation with peripheric embolism triggered a high index of suspicion of embolic mechanism in our patient and prompted a rapid assessment and successful management.

## 1. Introduction

Similarly to adult patients, when it comes to children, primary cardiac tumors have a scarce incidence of only 0.5% [1,2,3]. Most of them are primary tumors, originating from the heart, and histologically are proven to be benign. In contrast to the adult population however, where the most common types of cardiac tumors are myxomas, in the pediatric population, this type of tumor is rarely seen, with rhabdomyomas being the most frequently diagnosed [4]. However, cases of childhood myxomas have been published in [3,4,5]. When present, myxomas are typically localized in the left atrium [6], but there are few cases of left ventricular myxomas present in the literature [3,6,7,8,9].

Despite their benign nature, atrial myxomas usually present with either cardiac or extracardiac clinical manifestations. Failure to diagnose and properly treat them might lead to severe complications. In a review published by Castillo et al. in 2010 [10], numerous clinical presentations have been linked to the presence of a cardiac myxoma. Depending on its localization in the heart, either signs of right heart failure or left heart failure can be seen. Extracardiac complications such as tumoral embolisms have been reported. In addition to this, systemic inflammatory syndrome has been seen in patients diagnosed with a cardiac myxoma, due to tumoral cytokine release [7,10]. Although in most cases the patients are symptomatic, thus prompting further cardiac investigations, there have been cases of asymptomatic patients reported in the literature, where the cardiac mass was an incidental finding [3,7]. The presence of a mass in the left heart chambers might lead to either an inflow or outflow obstruction. Regarding extracardiac manifestations of myxomas, they can lead to systemic embolisms, which can be seen in around 50% of cases [7]. Another manifestation reported is systemic inflammation determined by increased tumoral secretion of interleukin-6 [4,10]. 

The preferred diagnostic method is usually transthoracic echocardiography, but thoracic computed tomography might be useful as well. Utilizing cardiac ultrasonography, myxomas can be seen as solitary hyperechoic masses of different sizes and localizations, and they are usually mobile and have a peduncular insertion [8,10,11]. A differential diagnosis with an intracavitary thrombus is mandatory, as it might determine the therapeutic strategy. 

Bearing in mind the fact that this type of tumor may lead to embolic complications, as well as potentially severe arrhythmias, early surgical treatment is required following the diagnosis of cardiac myxomas. The location of cardiac tumors usually determines the surgical approach [12,13]. The same applies to cardiac myxomas. In a study on adult patients with cardiac myxomas, Padalino et al. discussed the differences in postoperative prognosis depending on the localization of the cardiac tumors. Ventricular myxomas have been linked to the development of more complications than atrial tumors [14]. 

In this paper, we report the unusual case of an 11-year-old female patient with embolic bilateral lower leg ischemia, due to a highly unlikely left ventricular tumor, with a unique echocardiographic appearance.

## 2. Case Report 

An 11-year-old girl, currently in an 8-year remission from B-cell acute lymphoblastic leukemia diagnosed in her early childhood, presented in our emergency department for bilateral lower limb acute pain starting suddenly when in an apparently good healthy state. In the previous hours leading up to the onset of her symptoms, she performed moderate physical effort while rollerblading. During that activity, she felt a sudden pain in both her lower limbs, which was worse on the left side. When the pain was complicated by paresthesia and mild motor deficit, she was brought to the emergency department, about 6 hours after the onset of symptoms. 

Upon physical examination, the patient presented an antalgic position of the lower limbs, with spontaneous, intense pain, accentuated by passive movement, loss of skin sensitivity, paresthesia, and distal motor deficit. Moreover, her lower limb extremities were cold, pale, and slightly cyanotic, with an absent right femoral pulse and absent bilateral peripheral pulses. No other significant changes were noted during the physical examination. Aside from the acute pain, she was in a good general state. No other skin anomalies or lesions and no muscular or skeletal abnormalities were seen. She presented with no respiratory distress and was hemodynamically stable, without any recent history of acute illness.

The blood tests revealed a significant increase in muscle enzymes (CK-MB-165 U/L, CK = 15,789 U/L, LDH = 960 U/L) and mild anemia. The initial electrocardiogram (ECG) showed a normal sinus rhythm and high QRS voltage, while no signs of ischemic changes were visible. Due to the nature of the symptoms, our patient underwent both cardiac and arterial Doppler ultrasound. 

**Figure 1:**
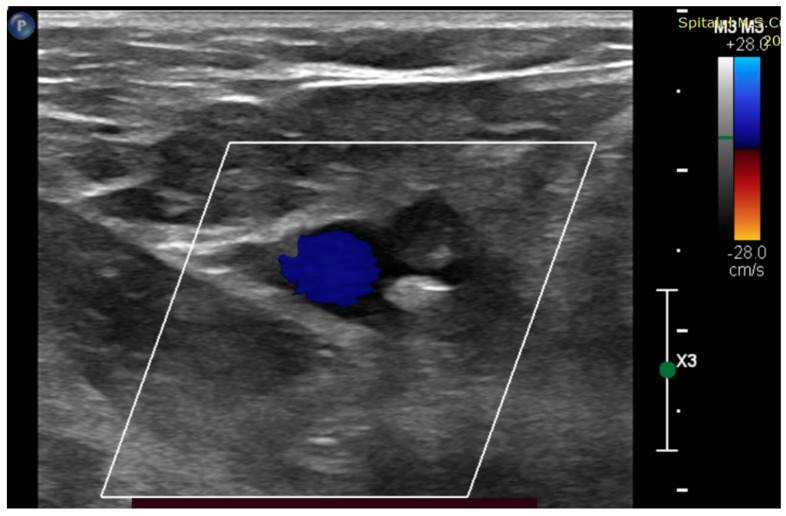
B—mode vascular echo image—short axis: right distal external iliac artery with an intravascular myxoma fragment.

**Figure 2:**
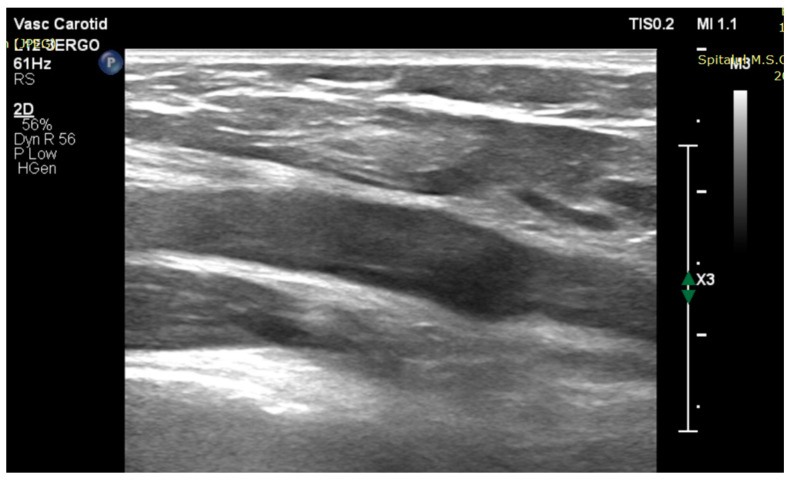
B-mode vascular echo image—long axis: right superficial femoral artery with occlusive tumoral fragment.

Bilateral lower leg ischemia suspicion was confirmed. On the right side, arterial Doppler ultrasound revealed subocclusion of the external iliac artery (Figure 1) and total occlusion of the femoral arteries (Figure 2). On the left side, the occlusion was localized in the proximal tibio-peroneal trunk. 

**Figure 3:**
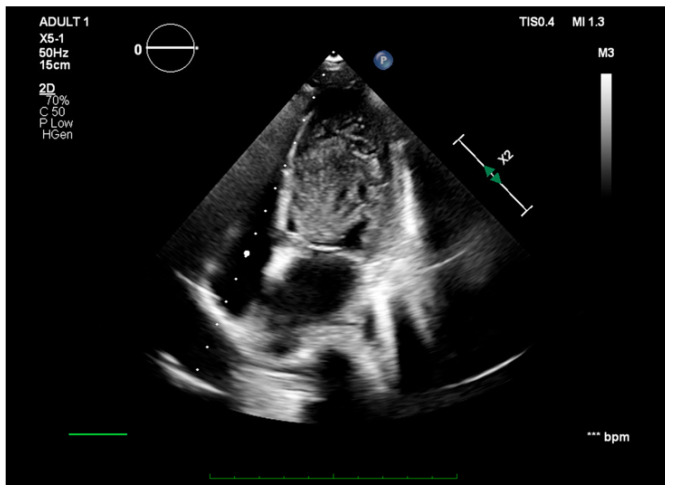
Apical four-chamber view showing a massive left ventricular tumor attached to the apical region and the interventricular septum.

**Figure 4:**
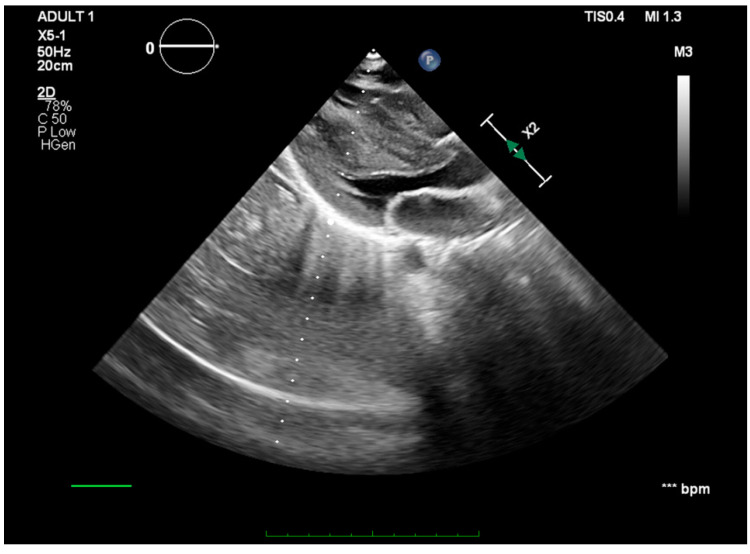
Parasternal long-axis view—the intracardiac tumor protrudes through the aortic valve during systole.

Cardiac sonography revealed a gigantic 6.3/3 cm, hyperechoic, unevenly defined left ventricular mass attached to the apical region and the interventricular septum (Figure 3). Due to its increased size and extremely mobile edges, the tumor was protruding through the aortic valve during systole (Figure 4). While no important aortic stenosis was present, a mild aortic regurgitation could be seen. The echocardiography showed no other significant changes. 

**Figure 5:**
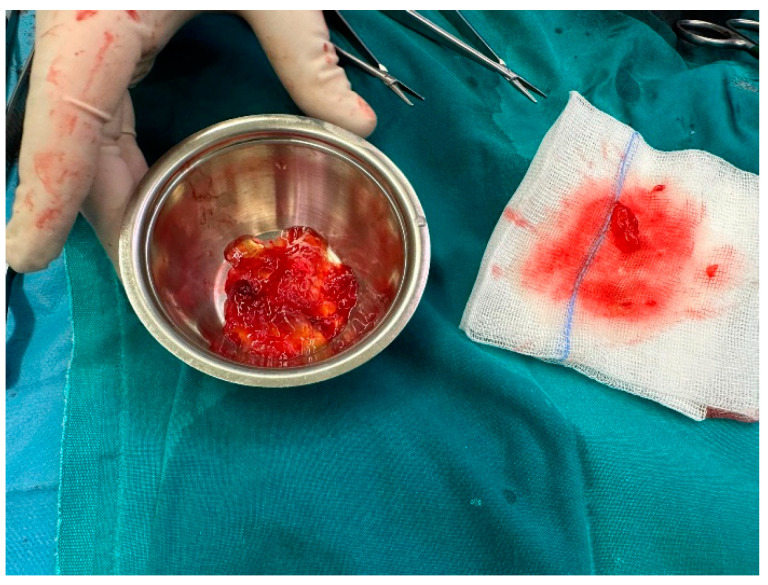
Excised cardiac tumor and tumor fragment embolus from the femoral artery.

In settings of bilateral acute lower limb ischemia confirmed by Doppler ultrasounds associated with a very friable gigantic left ventricle tumor, the embolic mechanism was the most likely. With over 8 hours since the onset of symptoms, we assumed that she presented with prolonged limb ischemia, which was endangering her functional abilities. So, bilateral embolectomy with Fogarty catheter and the removal of tumoral arterial obstructive fragments was performed on an emergency basis. Considering the high embolic risk of this hypermobile, fragile, cardiac tumor (and being especially concerned about the risk of stroke), an indication for tumor excision was established. So, after the embolectomy procedure, in the same surgical session, under cardio-pulmonary bypass, a giant, translucent, jelly-like consistency, left ventricular tumor was excised and sent for histopathological examination (Figure 5). The histopathological and immunohistochemistry reports were consistent with the diagnosis of an intracardiac myxoma.

**Figure 6:**
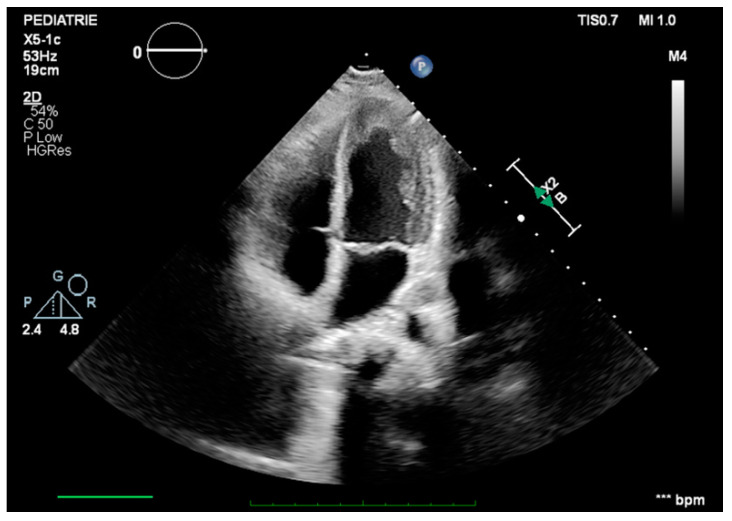
Apical four-chamber echocardiographic view during the early postoperative period, showing no residual tumor.

Following surgery, complete blood flow was restored in both of her legs and no residual intracardiac mass could be seen (Figure 6). Although our patient did not present suggestive symptoms for embolization in other areas, we still performed a thoracic and abdominal computed tomography angiography (on postoperative day 4), which showed no other signs of tumor embolism. From a clinical point of view, during the early postoperative period, our patient continued to present with pain and reduced sensitivity in both limbs associated with a motor deficit in the left lower limb extremity (with concordant electromyographical changes).

The evolution was slowly favorable and the patient was discharged after a two-week hospital stay, with improving neuromotor and neurosensory disorders and a recommendation of 3-month oral anticoagulant therapy and continuity of physical therapy. A 6-month follow-up showed no recurrence of the tumoral mass and complete recovery of her lower limb motor and sensitivity function. 

## 3. Discussion

We have presented a case of an eleven-year-old girl with a history of acute lymphoblastic leukemia (in remission for eight years), who developed a massive left ventricular cardiac myxoma and peripheral embolism with acute lower leg ischemia. 

Although a more common occurrence in the adult population, we also raised the suspicion of a cardiac myxoma with peripheric embolism, based on clinical presentation. 

A prompt diagnosis was made and an emergency surgery for both lower leg ischemia and cardiac myxoma was performed, which proved to be successful. 

A cardiac tumor is a rare occurrence in children [7]. Rhabdomyomas are the most frequent tumors seen in children, while myxomas arise in around 15% of cases [4] and are usually localized at the atrial level. Ever since 1960, when Chao et al. first described a case of cardiac myxoma in a pediatric patient [15], only a handful of cases of left ventricular myxomas have been published in the literature. 

Even though they are considered benign tumors, serious cardiac and extracardiac complications such as low cardiac output and systemic embolisms can occur [16]. There have even been cases of tumoral embolic strokes reported in the literature [17]. The patient in the case described by Chao et al. in 1960 also presented with an internal carotid embolic occlusion, in the context of a cardiac myxoma [15]. The case we presented also had systemic embolization but fortunately the affected territory (ischemic inferior limbs) could be saved with no residual damage due to emergency surgery. In our patient, the size and friability of the cardiac mass, as well as the fact that it was protruding through the aortic valve, were considered to be the main risk factors for recurrent embolism and the strongest argument for emergency surgical tumor excision. However, despite its size, the tumor did not determine decreased preload or significant outflow obstruction of the left ventricle, explaining the fact that before the embolic event, the patient was asymptomatic.

The literature data show that cardiac myxomas can be asymptomatic or may determine systemic inflammatory syndrome leading to non-specific symptoms such as fever, rash, weight loss, and gastrointestinal manifestations [7]; in this context, a diagnosis of a cardiac myxoma might be easily missed, leading to the progression of the disease. 

An interesting particularity in our case is the localization of the tumor, with left ventricle cardiac myxoma being the most rarely encountered. Zhao et al. reported in 2023 their 12 years of experience with 23 pediatric cases of cardiac myxomas; interestingly, none of the patients had a left ventricular mass.

Also, it must be emphasized that the crucial medical decision in our case was performing an embolectomy as soon as possible, without any delay for unessential investigations at that moment. The restoration of blood flow was obtained 10 h after the onset of ischemic symptoms and prolonging this time could have led to permanent functional damage of the lower limbs.

It should be mentioned that our patient was in remission after acute lymphoblastic leukemia and this is a very uncommon situation consisting of two unrelated rare diseases in the same patient. Unfortunately, the regular follow-ups carried out for oncologic diseases in her case did not include an echocardiography in the last 5 years. Although there have been adult cases of cardiac myxomas associated with a positive history of chronic leukemia [18], no such cases have been reported in patients with acute leukemia. Moreover, we found no pediatric cases reported in the literature where a cardiac tumor was diagnosed in a patient with a positive history for hematologic malignancies. To our knowledge, there is no literature data to support a possible correlation between the two conditions. 

However, considering the presence of cardiac myxoma in a child with a history of neoplasia, we took into consideration a possible Carney complex, which was excluded on a genetic testing basis [19,20,21].

Common postoperative complications in surgically treated cardiac tumors include valvular dysfunction, arrhythmic or infectious episodes, and pericarditis [1]. As stated above, there have been studies that have shown differences in postoperative prognosis in patients, depending on the tumoral location [14]. In these studies, ventricular tumors have been reported to present with more postoperative complications, due to ventricular wall resection. However, in the case that we have presented, the postoperative evolution was favorable and no significant complications were seen at periodic follow-ups. 

When it comes to recurrence, myxomas have a high rate due to partial resection [13]. Common risk factors leading to recurrence include incomplete resection and embolisms [20]. Our patient did not present any signs of recurrence at the 6-month follow-up, but we consider that in this case long-term surveillance is needed. 

## 4. Conclusions

We have presented a rare case of a massive left ventricular myxoma complicated with bilateral lower limb systemic embolism in an 11-year-old patient. Tumors of this size, aspect, and localization have been scarcely represented in the literature, especially in the pediatric population. Despite its size and systemic complications, following emergency surgical treatment after prompt diagnosis, our patient’s evolution was uneventful, with no recurrence of the intracardiac tumor and no residual symptomatology. 

## Data Availability

Data available upon request.
